# Survival, healing, and swim performance of juvenile migratory sea lamprey (*Petromyzon marinus*) implanted with a new acoustic microtransmitter designed for small eel-like fishes

**DOI:** 10.1186/s40317-023-00318-1

**Published:** 2023-03-11

**Authors:** Taylor F. Haas, Theodore Castro-Santos, Scott M. Miehls, Zhiqun D. Deng, Tyler M. Bruning, C. Michael Wagner

**Affiliations:** 1 Department of Fisheries and Wildlife, Michigan State University, 13 Natural Resources Building, East Lansing, MI USA; 2 U.S. Geological Survey, Eastern Ecological Science Center, S.O. Conte Research Laboratory, Turners Falls, MA USA; 3 U.S. Geological Survey, Great Lakes Science Center, Hammond Bay Biological Station, Millersburg, MI USA; 4 Pacific Northwest National Laboratory, P.O. Box 999, Richland, WA USA; 5 Department of Mechanical Engineering, Virginia Tech, Blacksburg, VA 24061 USA

**Keywords:** Lamprey, JSATS, ELATS, Acoustic Telemetry, Survival, Burst Swim, Endurance Swim, Wound Healing, Surgery

## Abstract

**Background:**

Little is known about the transformer stage of the parasitic lampreys, a brief but critical period that encompasses juvenile out-migration from rivers to lakes or oceans to begin parasitic feeding. Information about this life stage could have significant conservation implications for both imperiled and invasive lampreys. We investigated tag retention, survival, wound healing, and swim performance of newly transformed sea lamprey (*Petromyzon marinus*) implanted with a new micro-acoustic transmitter, the eel–lamprey acoustic transmitter (ELAT), in a controlled laboratory environment.

**Results:**

The 61-day survival of our tagged subjects was 71%, within the range reported in similar studies of juvenile lampreys. However, survival was significantly lower in the tagged animals (vs control), with no effect statistically attributable to measures of animal length, mass, condition, or population of origin (Great Lakes vs. Atlantic drainage). Mortality in tagged fish was concentrated in the first four days post-surgery, suggesting injury from the surgical process. An unusually long recovery time from anesthesia may have contributed to the increased mortality. In a simple burst swim assay, tagged animals swam significantly slower (− 22.5%) than untagged animals, but were not significantly different in endurance swim tests. A composite wound healing score at day four was a significant predictor of maximum burst swim speed at day 20, and wound condition was related to animal mass, but not length, at the time of tagging.

**Conclusions:**

Impairments to survival and swim performance of juvenile sea lamprey implanted with the ELAT transmitter were within currently reported ranges for telemetry studies with small, difficult to observe fishes. Our results could be improved with more refined anesthesia and surgical techniques. The ability to track migratory movements of imperiled and pest populations of parasitic lampreys will improve our ability to estimate vital rates that underlie recruitment to the adult population (growth, survival) and to investigate the environmental factors that regulate the timing and rates of movement, in wild populations.

## Background

Migration is a time of heightened threat and uncertainty regarding the risk of predation and the localization of critical resources [60]. This is particularly so when migration occurs early in life, when variation in daily growth and survival may be substantial and influence recruitment to the adult population [24, 37]. A number of economically and ecologically valued fishes exhibit juvenile feeding migrations from rivers into lakes or oceans, including salmon (*Oncorhynchus* spp.) and lake sturgeon (*Acipenser fulvescens*). Threats to the viability of these populations, and to control invasive species with similar life histories, have prompted considerable interest in ascertaining the behavior, timing, and survival of juvenile fishes as they move downstream [35, 56].

Several lamprey species are among the most poorly understood fishes that exhibit juvenile migration, including species that range from imperiled and ecologically or commercially valued (e.g., Pacific lamprey *Entosphenus tridentatus*, European river lamprey, *Lampetra fluviatilis,* sea lamprey *Petromyzon marinus* in select European regions) to pestilential and targeted for reduction (e.g., invasive sea lamprey in the Laurentian Great Lakes). These lampreys spawn in rivers, after which newly hatched larvae (ammocoetes) bury in stream sediments for typically four to seven years feeding on organic detritus and algae [14]. At the end of the larval stage, they transform and migrate downstream into estuaries or large lakes to commence parasitic feeding on fishes [1, 14, 21]. This period of physiological and geographical transition (aka the transformer stage) [9] is of short duration, yet is considered critical to the development of effective conservation and management practices [21]. For example, out-migrating lamprey within their native ranges typically must transit through a gauntlet of dams and their affiliated water intake structures, resulting in physical injury, increased predation, and direct or delayed mortality [27, 48]. Providing for safe passage through these structures is necessary for the protection of the species [26].

In the Laurentian Great Lakes, where a single sea lamprey may consume upwards of 21 kg of fish biomass, control is achieved through the application of selective pesticides (lampricides) to kill larvae in streams prior to transformation [5, 43]. Rivers are selected for lampricide treatment based on estimates of larval abundance, informed by population models and expert opinion [34]. The ability to estimate the fate of out-migrating sea lampreys would allow treatment decisions to be made based on probable parasite production vs. larval abundance, incorporating system-specific differences in rates of survival through migration and first feeding. For example, in Lake Michigan, the lower reaches of rivers systems often contain drowned river mouth lake–wetland complexes that are rich in piscivorous predators [38], and piscivores are important predators of out-migrating juvenile lampreys [48, 58, 62]. Life stage-specific spatial population models for sea lamprey currently assume all out-migrating sea lamprey have an equal likelihood of survival until arrival in the lakes, regardless of differences in migratory distance, river size, habitat types, or predator populations, each of which contribute to differences in mortality and growth among watersheds [31, 59].

Survival estimation during migration requires tracking an animal’s status over considerable distance and/or time. The most frequently used tool to monitor the movements of fishes and other aquatic organisms over large distances is telemetry, involving the implantation or attachment of a transmitter that may be detected when the fish moves into the range of a receiver [32]. Until recently, small (≤ 12 mm) passive integrated transponder (PIT) tags were the only transmitters available for the study of out-migrating lamprey. PIT tags lack an internal power source, requiring tagged fishes to move within 1–2 m of the antenna to be detected [3, 53]. The proportion of PIT-tagged larval and out-migrating lamprey detected by PIT antennas is relatively low, with reported detection rates ranging from 5 to 14% [13, 47]. Internally powered telemetry tags (acoustic and radio transmitters) offer an alternative that substantially increases the detection range (tens to hundreds of meters) and detection probability (as high as 80–100% on a well-designed array covering the entirety of the stream channel) at a single receiver [45]. Acoustic transmitters have several desirable properties for use in small fishes, in particular, they do not require an external trailing antenna (as radio telemetry typically does), that may result in negative impacts to swim performance, lower likelihood of predator attack, and higher survival in tagged individuals (reviewed by Crossin et al. [11] and references therein).

A primary assumption of telemetry analysis is the fish’s movement behavior (e.g., swim speed, timing) is not substantially altered by bearing the tag, or because of the implantation process. Historically, acoustic transmitters have proven too large to implant into small fishes with narrow body cavities. Transmitters with sufficient battery life to support demographic studies of fishes require large batteries that would impede movement and survival in small fishes (Liedtke 2019). This situation is compounded in anguilliform swimming fishes, where tags may physically impede propagation of the propulsive wave or compress organs along the length of the very narrow body. The fabrication of a novel microbattery and transmitter suitable for use in juvenile sea lamprey—the Eel–Lamprey Acoustic Tag or ELAT—offers a potential solution to this problem [15, 39]. This tag is compatible with the JSATS (Juvenile Salmon Acoustic Telemetry System) receiver, with a detection range of 80–140 m, a distance 100 × greater than the approximate range of a 12 mm PIT tag [17]. The transmitter has a source level of a 147 dB and is in a cylindrical encasing with dimensions 12 mm × 2 mm, 0.08 g dry-mass and transmits at a frequency of 416.7 kHz (± 0.5%). Currently, the standard PRI (Pulse Rate Interval) of five seconds allots approximately 30 days of use [16]. Pilot field studies using the ELAT tag have demonstrated the high detection rates (> 95%) in juveniles of both Pacific lamprey and American eel (*Anguilla rostrata*) [17, 39]. At approximately the same size as the PIT tags used in previous studies of juvenile lampreys (12 mm x 2 mm, 0.08 g), this is the first acoustic telemetry tag that does not persistently violate the two percent of body mass standard for use without impediment of movement in juvenile anguilliform fishes due to its cylindrical shape [46, 53, 63]. Here, we report an assessment of tag retention, survival, wound healing, and impacts to swim performance of the ELAT transmitter surgically implanted into newly transformed sea lampreys. Our goal was to perform a comprehensive evaluation of the impacts of ELAT transmitter implantation to support the establishment of criteria for use in field studies designed to enumerate critical demographic parameters (e.g., stage-specific mortality rates) and the movement ecology of the poorly understood transformer stage.

## Methods:

### Study design

The objective of this study was to examine the effects of the ELAT tag on sea lamprey survival, condition, and swim performance. To achieve this, we surgically implanted ELAT tags into 59 newly transformed juvenile parasitic sea lamprey and examined tag retention, survival, wound healing, and swim performance during a 61-day study period, corresponding to anticipated tag life at a PRI of 10 s. Wound healing assessment involved two scoring metrics, wound closure and wound inflammation, and a composite score that combined the two measures into an overall measure of wound condition. Swim performance was analyzed by comparing two performance metrics, maximum burst swim speed (20 days post-surgery) and time-to-exhaustion swimming (29–31 days post-surgery), to untagged control fish. We focused on the animal’s burst swim ability, as it is a facultative behavior suggestive of ability to escape a perceived threat, and swim-to-exhaustion, as it is characteristic of long stretches of active swimming during a migration [19]. The swim measures were separated by ten days to ensure the stress of the burst swim test did not influence the results of the time-to-exhaustion test. All measures were statistically compared to a group of 54 control animals that did not experience the anesthesia, surgery, or tag implantation. Due to the difficulty in acquiring this life history stage in sea lamprey, subjects were collected from both Great Lakes and Atlantic basin drainages. Where appropriate, source was included as a covariate in the statistical models.

### Collection and housing of subjects

Sea lamprey used in this study were collected from tributaries in the Great Lakes Basin (*N* = 64), and a hydropower diversion canal on the Connecticut River that flows into the Atlantic Ocean (*N* = 49).

**Fig. 1:**
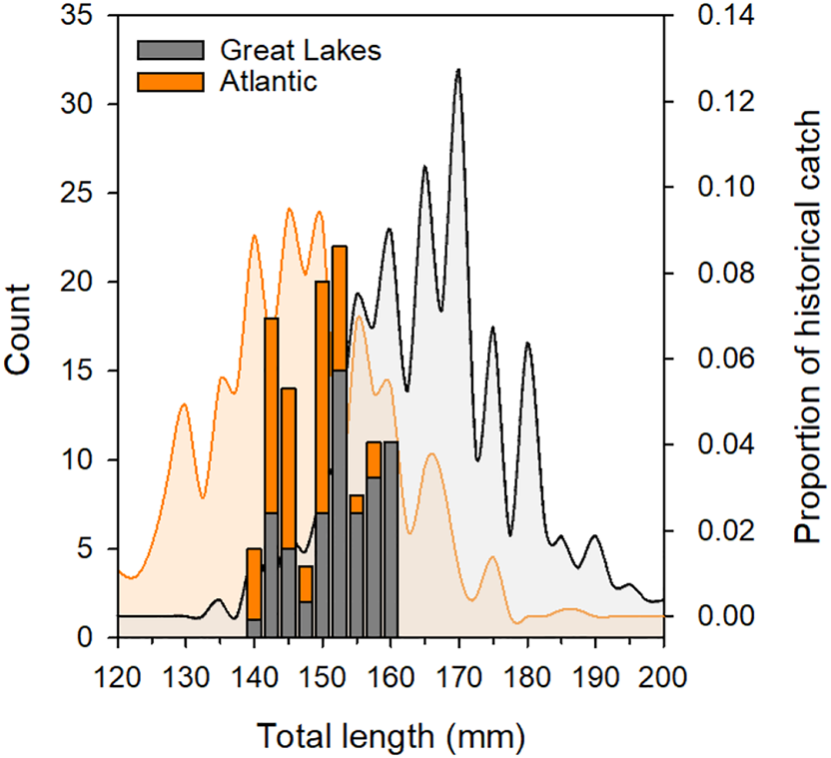
Density plots of total length (mm) for historic collections of out-migrating juvenile sea lamprey for animals captured from Great Lakes streams (Gray, *N* = 267) and Atlantic drainages in the northern United States (red, *N* = 653) are overlaid with a stacked histogram representing the two harvest locales and illustrating the experimental subjects total length (Great Lakes: *N* = 59, Anadromous: *N* = 54). Data for historical collections were compiled by J. Hume, Michigan State University

We selected 113 sea lamprey ranging 140 mm–160 mm total length (TL) based on recommendations from previous studies using similarly sized PIT tags in juvenile sea lamprey [63] and Pacific lamprey [46]. We chose to include animals across a range of sizes near the median of the size distributions in the Great Lakes and the Atlantic basin (Fig. 1) to examine the relationship of body size to wound healing and swim performance.

Subjects were housed at the U.S. Geological Survey Hammond Bay Biological Station from February 11, 2020 to April 13, 2020. Lampreys were separated by source and kept in eight separate 23L 40 cm x 22 cm x 26 cm tanks that were supplied with constant flow-through water from Lake Huron and continuous aeration. As our findings are intended to inform use of the ELAT tag in natural rivers, the holding tanks were constructed to include certain natural features the animal would experience during out-migration. First, previous tagging effects studies observed fungal growth and infection on experimental subjects held in the laboratory [7, 49, 50]. To ensure the microbiome the sea lamprey were exposed to was akin to the natural flora, we lined the tanks with a 4 cm layer of mixed substrate (cobble, sand, fine-grained sediment) collected from nearby Schmidt’s Creek. In addition, unfiltered inflowing water from Lake Huron was heated to a temperature typical of central Lake Michigan tributaries during the fall migration (~ 4.5° C).

### Surgical implantation of the transmitter:

ELAT transmitters were fabricated at the Pacific Northwest National Laboratory by Daniel Deng. This experiment used a modified ELAT where the microbattery was replaced with a PIT transmitter to allow for individual identification of the subjects via a hand scanner. The measured dimensions of the modified tags used in this study were as follows (mean ± 1 SD): weight in air, 0.0809 ± 0.003 gm; length, 12.07 ± 0.10 mm; and width, 0.20 ± 0.003 mm. On February 12, 2020, 59 fully transformed sea lamprey (per [76]) were surgically implanted with modified ELAT transmitters (30 Great Lakes and 29 Atlantic drainage), and 54 entered the experiment as control animals (34 Great Lakes and 20 Atlantic drainage). Animals in the tagged group measured (mean ± 1 SD) 150.16 ± 5.69 (total length, mm) and weighed 4.37 ± 0.64 g. Animals in the control group measured (mean ± 1 SD) 148.30 ± 6.08 (total length, mm) and weighed 4.18 ± 0.50 g.

The implantation surgery followed protocols established by Moser et al. [49] and Christiansen [7]. An anesthetic bath of AQUI-S-20E (10% eugenol) and lake water was prepared to sedate the animals undergoing the tagging procedure. Anesthesia concentrations from prior studies (0.02–0.06 ml l^−1^, USFWS 2013, Simard 2017) proved ineffective at inducing stage-IV anesthesia in pre-surgery screening [66]. Similar occurrences have been noted for other anesthetic agents applied to juvenile lamprey when using doses recommended for teleost fishes. Stage-IV anesthesia was induced in (mean ± 1 SD) 17.0 ± 4.5 min via immersion in a solution of 0.7 ml l^−1^ AQUI-S-20E (eugenol concentration = 70 ppm). When each animal reached stage-IV anesthesia, it was placed on a damp surgery board and the surgeon (T.F. Haas) made a lateral 3 mm incision into the body cavity adjacent to the 13th myomere using a Premier Edge Restricted Depth 3 mm microscalpel (OASIS^®^ Medical). A disinfected (immersion in 70% ethanol for ≥ 2 min) then rinsed transmitter was inserted posteriorly into the opening until it was completely enveloped in the animal’s body cavity (mean ± 1 SD = 1.4 ± 0.93 min, range = 0–4 min); no suturing or other method of artificially closing was used [13, 14, 49]. After the transmitter was fully inserted, each animal was placed into an oxygenated recovery tank until active swimming or suction attachment to the side of the tank was observed, indicating initial recovery (mean ± 1 SD time to recovery = 102.08 ± 67.13 min, range = 18–414 min). After recovery, the subjects were returned to their holding tanks for the experimental phase.

### Mortality, wound healing, and condition:

Mortalities for both tagged and control treatment groups were enumerated daily. Wounds were visually evaluated by a single individual (TFH) at days 4, 20, 29, 44, and 61 for all animals alive and retaining a transmitter at the time of observation. For evaluation, animals were removed from their tank and individually placed in a small, clear, tank where a photo was taken when the animal ceased movement. No anesthesia was used. Control animals were not examined assessment criteria were modified from that of Wagner et al. [72] and Moser et al. [49], where wound closure and wound inflammation were scored separately on a scale of 1–4 (Table 1). An additional metric, a composite wound score, was produced from the wound closure and wound inflammation scores. This was formulated by plotting the wound closure and wound inflammation scores on an X–Y plane (axes range from 0 to 4) and measuring the Euclidean distance from the origin to the observed scores (Eq. 1). To ease interpretation, that distance was then rescaled to a range of 0–10, based on the minimum (1.41, i.e., wound closure and inflammation scores of 1) and maximum (5.657, i.e., wound closure and inflammation scores of 4) possible distances from the origin:

1 $$\left( {\frac{Euclidean \,Distance - \sqrt 2 }{{\left( {\surd 32 - \surd 2} \right)/10}}} \right).$$

**Table 1:** Criteria for assessing wound closure and wound inflammation. Modified from Moser et al. [49] and Wagner [72]

Score	Wound closure	Wound inflammation
1	Severed tissue is rejoined and completely healed	No inflammation or discoloring internal or external to the wound
2	Tissue is apposed but remains severed	Slight gray or pink tissue internal or external to the wound is present. Organs completely internal
3	Portions of the wound are apposed. This may include apposition around a protruding tag	Some tissue internal and external to incision is inflamed or discolored (gray or red). May also be characterized by intestines partially protruding through incision site
4	No severed tissue is apposed	All tissue internal and external to wound is inflamed or discolored (gray or red). May also be characterized by intestines completely protruding and external from body cavity through incision site

### Swim performance

Twenty days after implantation and after the second wound healing assessment, we measured burst swim velocity in all implanted and control animals remaining in the study. Animals were placed in a 155 × 13x10cm plastic trough filled with 8 cm of water at ± 1 °C of the holding tank temperature. A plastic mesh grid marked in 5 cm intervals was positioned in the bottom of the trough to measure distance moved, and all interior surfaces were covered with plastic mesh to prevent animals from attaching to the trough surface. Each animal was placed in the lower end of the trough and allowed a five-minute acclimation period prior to testing. After the acclimation period, maximum speed was measured by inducing a fast-start (“startle”) response. This was done by squirting approximately 3 ml of water from a 5 ml pipette at the water surface above the animal’s head [12, 50]. Each animal underwent three burst trials with a 3-min recovery period in between trials. A Go-Pro camera (60 frames/sec.) was mounted above the trough to record trials, and video footage was processed and analyzed using Kinovea^®^ motion analysis software. Burst speed was measured as the distance the animal traveled in the initial 30 frames (0.5 s) immediately following the first frame showing ripples produced by the pipette ejection. The maximum observed speed (cm s-1) of the three trials was used in analyses below, hereafter referred to as an individual’s burst speed.

On days 30 through 32 post-surgery, all implanted animals remaining in the study (n = 30) and 32 control animals chosen haphazardly from approximate same source proportions were subjected to an endurance swimming test (time-to-exhaustion) in a swim tunnel. Animals were individually placed in a 48.2 L plastic mesh-lined chamber of a 121 L Blazka-type swim respirator, with a Leeson Washguard Adjustable Speed AC Motor and Controller. The impeller was powered to 4.5 Hz. This speed was chosen as a result of preliminary trials where lamprey outside of the experiment were placed in the swim chamber and subjected to various velocities, with the optimal velocity being chosen as swift enough to (A) induce swimming and (B) prompt the cessation of swimming within several minutes. The inner-mesh lining of the swim tunnel was necessary to prevent subjects from attaching to the side of the tube, but rendered the manufacturer’s regression equation linking power units of the swim-tunnel impeller to water velocity imprecise. COVID-19 restrictions prevented empirical water velocity calibration; however, passive particulates were moving through the tunnel at ~ 15 cm s^−1^ (approximately 1 body lengths/s for our subjects). Consequently, this assay was used to compare differences between groups, not to precisely estimate time-to-exhaustion in tagged and untagged sea lamprey.

After a 3-min acclimation period with no water flow, animals were induced by water flow to freely swim against a current. As the test progressed, animals would become impinged on the mesh barrier at the downstream end of the tunnel. While the animal maintained position in the flow, actively swimming against the current, elapsed time was recorded. If the animal became impinged, but maintained active swimming motions (tail-beating) resisting the current, elapsed time continued to be recorded. In the first instance an animal became impinged with a continued absence (10 s) of anguilliform movement resisting the water current, a Smith-Root backpack electro-shocking unit sent a brief 12 V, 1 Hz, and 5% duty cycle electrical current into the rear of the swim tunnel for up to ten seconds. This mild voltage was intended to irritate rather than stun the animal so swimming could recommence with full musculature control. Trials continued for animals induced to resume swimming within ten seconds of continuous electrical current. We deemed exhaustion occurred (trial completed) when either of two events occurred: (1) an absence of anguilliform swimming for ten seconds after the second impingement, or (2) no resumption of active swimming within ten seconds of electrical current after the initial impingement. If the animal continued to swim for 60 min without exhaustion, the trial was ended.

### Data analysis (tag retention and survival)

Tanks were monitored daily for shedding of transmitters and mortalities by visually searching the substrate for transmitters and immobile animals. No burrowing activity was observed during this experiment; therefore, a visual inspection of the surface ensured that any mortalities or shed tags were seen and that the animals were minimally disturbed. Immobile animals were tapped with a net to determine if the subject was non-responsive. Dead animals and shed tags were removed from tanks and recorded daily until the end of this study.

Daily mortality data recorded from a 61-day holding period were used to generate Kaplan–Meier survival curves for control and tagged animals; a log-rank test (Mantel-Cox) was used to assess the difference in survival among the two groups. The *p-*value resulting from a Log-Rank test determines the level of significant difference in survival between tagged and control groups for the entirety of the experiment. This potentially creates “blind spots” in the analysis, which would prevent the identification of critical periods when large changes in mortality occurred between groups (e.g., immediate post-surgical or post-swim testing mortality). These periods have high informative value for future studies, especially for field studies seeking to use this technology (e.g., setting the post-surgical holding period if surgery related mortality is delayed by a few days). We further explored the data to determine if and when any blind spots occurred. To do this, we repeatedly subset the survival data, creating a new dataset for each day (day_i_) including data from day_*i*_ until the end of the experiment. This nullified deaths prior to day_*i*_, thereby establishing day_i_ as day 0 in the analysis for each dataset (i.e., the first subset equals the whole dataset, from day 1 onward, the second contains only day 2 onward, etc.). A Log-Rank test of each dataset produced *p-*values for the period following day_i_ that were evaluated by generating Kaplan–Meier survival curves. This progressive *p-*value provided insight by illustrating the pattern of the significance level in the difference between the tagged and control groups survival through time. These *p*-values were used for qualitative visualization only, with statistical significance hinging on the full dataset log-rank test.

Upon reviewing the survival curves of the tagged and control groups, it was apparent there was a time-dependent component in the survival curves. Mortalities appeared to occur in punctuated events within a group (i.e., were not homogeneous throughout the 61-day holding period). Kaplan–Meier-based survival analyses restricted to only one covariate are unable to capture this effect. Therefore, additional analyses were performed using Cox-Proportional Hazard (Cox model) regression (R package “survival” v.2.44–1.1, [69, 70]) with a time-dependent covariate (TDC) to determine if tag implantation was associated with an increased risk of mortality, and to what extent differences changed over time. The time-dependent covariate was a binomial categorization that was set to one for tagged and zero for control animals surviving to Day 32, which gave the model an explicit value to measure the significance and magnitude of a tag effect from days 32 to 61. This model was compared using Akaike Information Criterion (AIC) and heteroscedasticity of the Schoenfeld residuals to a null model of one covariate (Group) to assess the role of the TDC in the model’s fit. We used a counting process form data frame to build the TDC into the dataset, constructed using methodology similar to that of Zhang et al. [78] and Therneau [70]. An individual animal is represented by one row if mortality was experienced prior to Day 32 and two rows if mortality was experienced after Day 32 or the animal survived the experiment in entirety (covariate set to 1 if tagged, 0 if control). Day 32 was chosen as the break point as it coincided with the first mortality event recorded in the control group. Schoenfeld residuals were examined to determine if the assumptions of a proportional hazard model were met. In both the Kaplan–Meier and Cox-Proportional Hazard analyses, tagged animals that shed their tag were not right-censored at the time of the shed event to preserve statistical power through sample size. Finally, animals experiencing early mortalities (< 5 days) were compared to animals surviving > 5 days through separate logistic regression with the three size metrics as predictors. Source (Great Lakes vs. Anadromous) was the additional covariate.

### Data analysis (wound healing and condition)

The two wound healing scores, and the composite score, were assessed through time via separate non-parametric Friedman tests, with the assessment score as the dependent variable and numeric assessment (1–5) as the predictor. Post hoc Wilcoxon pairwise rank sum tests using Bonferroni correction explored the significance of relationships between wound scores and numeric assessment [55]. Relationships between each of three size measurements (Total Length in mm, mass in g, and Fulton’s Condition Factor; Eq. 2; Ricker [57]) and each wound score were explored through simple linear regression [55]:

2 $$\frac{mass(g)}{{Total \,Length(mm)}^{3}* {10}^{6}}.$$

### Data analysis (swim performance)

A two-way unbalanced ANOVA [22] was performed to determine the relationship between maximum observed burst speed and treatment group (Control vs. Tagged) with source (Great Lakes vs. Anadromous) as a covariate. Separate simple linear regression models determined the relationship of size and composite wound score to maximum burst speed for the tagged animals. A time-to-event analysis was used to compare the tagged and control group’s time-to-exhaustion probabilities by evaluating their respective Kaplan–Meier survival curves via log-rank test (R package “survival” v.2.44–1.1, [70], survival = continuing to swim). Plotted through time (log-seconds) on the x-axis, survival probabilities dropped on the y-axis as members of the respective groups reached their time of exhaustion. The log-rank test assessed the statistical difference between the cumulative exhaustion probabilities of each group. To assess significance, the log-rank test statistic, where the expected value is the product of the risk of event (number of exhaustion events/number unexhausted) and the number of unexhausted animals at the given time, is compared to the critical value (chi-square) for one degree of freedom [25]. Additionally, an unbalanced two-way ANOVA tested the null hypothesis that there was no significant difference between swim-to-exhaustion time and covariates group and source.

## Results

Four days into the experiment one holding tank containing 14 tagged and 1 control animal from the anadromous tank experienced 24 h without inflowing water or aeration. These animals were used in analyses prior to that point and censored from any analysis after that date [41].

### Tag retention and survival

Eleven of the 59 tagged animals (19%) shed their tags throughout the experiment. Two shedding events occurred when handling animals for endurance swim performance tests. The majority (5/9) of the remaining shedding events occurred within the first four days post-implantation, and all animals that shed their tags had wounds that were completely open with no apposition in the initial wound assessment on Day 4 (wound closure score = 4). The four additional shedding events were recorded on days 19, 20, and 29 (two individuals).

**Fig. 2:**
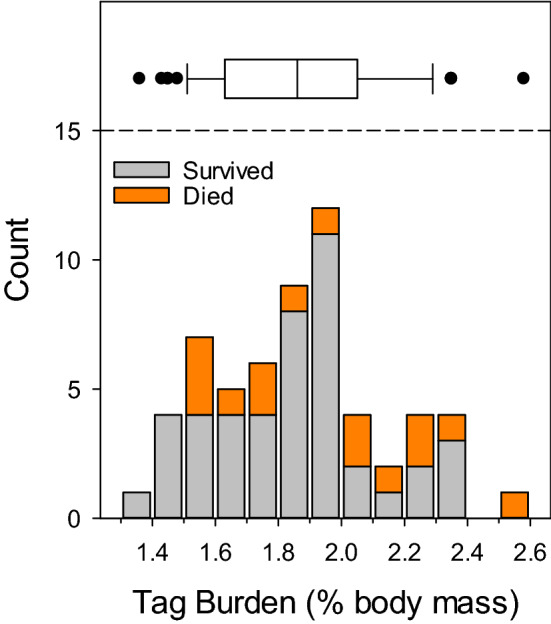
Frequency histogram of tag burden in a group of 56 sea lamprey fitted with an ELAT transmitter. Animals that died (*n* = 17) are indicated in orange. The upper box plot reports the median (vertical line), 25/75th percentiles (box), and 10/90th percentiles (whiskers) with outliers (filled circles)

Twenty-five sea lamprey died during the experiment (8 in the control group, 17 implanted with ELAT transmitters). The tagged group experienced increased mortality at the commencement of the experiment, with five animals dying within four days post-surgery (Days 1, 1, 2, 4, 4). Tag burden (tag weight/body weight, %) averaged 1.87 ± 0.036 across the entire tagged group, with mortalities distributed across the range (Fig. 2), but higher in animals with a tag burden above 2% (6/15, 40% mortality as a group) vs. those at or below 2% (11/44, 25% mortality as a group). However, no size metric (TL, Mass, Condition Factor) nor source was a significant predictor of early mortality (logistic regression, all *p*-values > 0.1). No mortalities were observed in the control group until Day 31, with a total 8 mortalities accumulating gradually over the following 25 days.

**Fig. 3:**
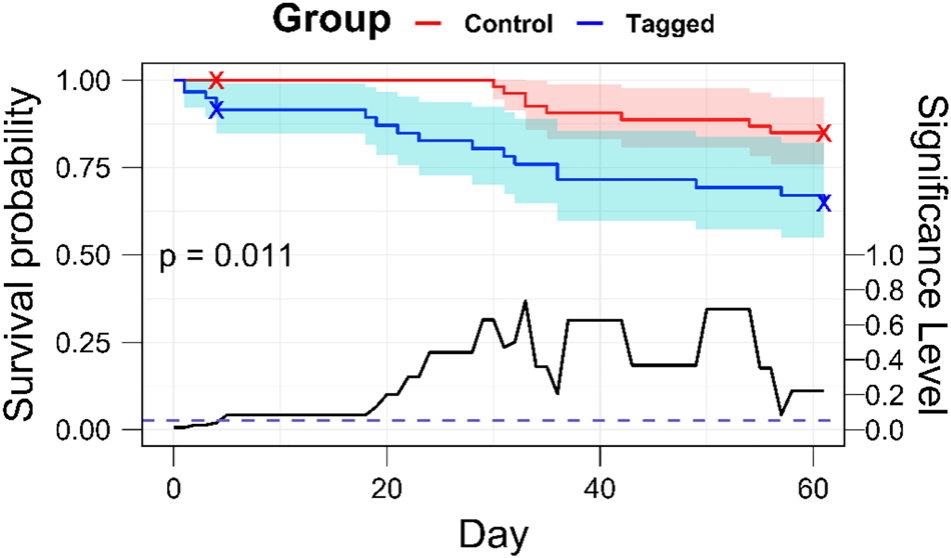
Kaplan–Meier survival curves with 95% confidence intervals for control (red) and tagged (blue) groups with the overall significance (Mantel-Cox log-rank test). A (x) symbol denotes a right-censorship event in the respective group. The progressive P-value (black line, right Y-axis) portrays the significance level associated with Log-Rank tests throughout each day of the experimental period. Each point on the line corresponds to the p-value for a Log-Rank test, adjusting the experimental period to begin on that day. The purple dashed line represents a significance level of 0.05

The cumulative survival probabilities (mean ± 95% CI) were 0.849 ± 0.092 and 0.647 ± 0.124 for the control and tagged groups, respectively. The observed difference in survival was significant (Mantel-Cox log-rank test, p = 0.011). Examination of the Kaplan–Meier survival curves and the progressive *p*-value (Fig. 3) suggests distinct periods of elevated mortality in both groups. The principal differences in survival between tagged and control groups appear in the four-day period immediately post-surgery (whereafter, the progressive *p*-value loses statistical significance), and Days 18–30, roughly corresponding to the period following the burst swim tests (Day 20, Kaplan–Meier curves, Fig. 3). Prior to date of the swim-to-exhaustion test (Day 30), no mortalities were recorded in the control group. Following this test, both groups exhibited similar reductions in survival. The Cox-Proportional hazard model suggested tagged animals were 7.4 times more likely to experience mortality in the first 32 days of the experiment vs. control animals (coefficient = 2.00, Hazard ratio = 7.4, *p* = 0.008). The time-dependent covariate coefficient was − 1.80 (*p* = 0.065, 95% CI -3.7—0.11, se = 0.97) which implies that after 32 days, the hazard ratio in the tagged group was reduced to of 1.22 $\left({e}^{2.00-1.80}\right)$. The ΔAIC (1.86) relative to the null model supports the assertion that the difference of associated risk through time between the two groups is not homogeneous. Moreover, the global Schoenfeld residuals of the time-dependent covariate model (*p* = 0.63) present less heteroscedasticity than those of the null model (*p* = 0.10).

### Wound healing and condition

**Fig. 4:**
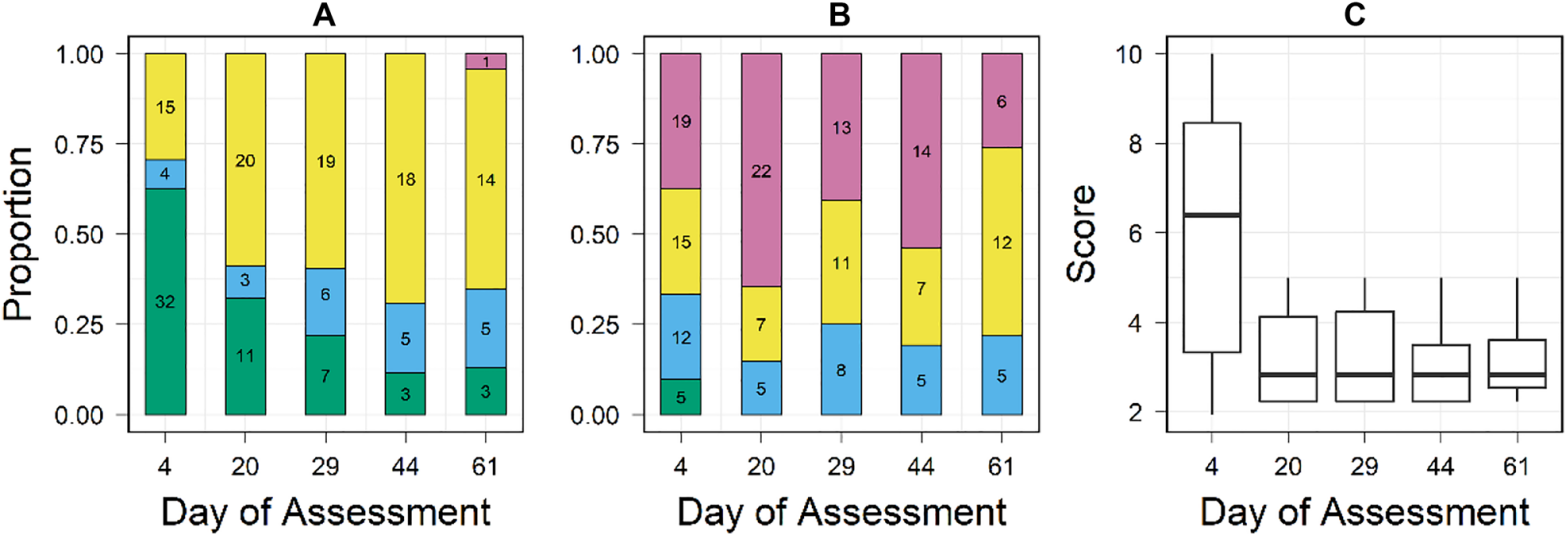
Wound assessment scores. **A** Distribution of wound closure scores for animals remaining in the study at the time of assessment. **B** Distribution of wound inflammation scores for animals remaining in the study at the time of assessment. **A** & **B** *1* = *Violet, 2* = *Yellow, 3* = *Blue, 4* = *Green* **C** Box plots of composite wound scores for animals remaining in the study at the time of assessment, showing median (black line), and maximum and minimum values (lines)

No animals received a composite score of 0 indicating a completely healed wound by the end of the study period (Fig. 4). One animal had a completely closed wound (wound closure = 1) on the final wound assessment, but mild inflammation was present (wound inflammation = 2). The initial composite wound score (Day 4) was unrelated to total length (separate univariate linear regressions: *p* = 0.07, Adjusted *R*^2^ = 0.04, SE = 0.06) or condition factor (*p* = 0.27, Adjusted R^2^ = 0.005, SE = 2.5) but negatively associated with mass (*p* = *0*.02, Adjusted *R*^2^ = 0.08, SE = 0.5). Each additional gram of mass was associated with an improvement of 1.2 composite score units (i.e., a 12% improvement in wound condition). Wound closure scores improved throughout the experimental period, with the greatest improvement occurring between the first and second observation dates (Days 4 and 20, post-surgery, mean change = 0.47 score units ± 0.24). Animals surviving the full experiment showed a slight decline on average in wound inflammation from Days 4 to 61 (0.17 score units ± 0.15), but inflammation scores improved between Days 4 and 20, with the same mean as wound healing. A Friedman rank sum test revealed time was a significant predictor for each of the wound scoring metrics (Table 2). Post hoc pairwise Wilcoxon Rank Sum Tests revealed a significant improvement in wound scores performed after Day 20 (Table 2).

**Table 2:** Cumulative wound scores (closure, inflammation, composite) Friedman Rank Sum comparison through time, with total *p*-values and those of pairwise Wilcoxon Rank Sum Tests between successive assessments

Assessment Type	Total *p*	*p*: Day 4 vs. Day 20-	*p*: Day 20 vs. 29	*p*: Day 29 vs. Day 44	*p*: Day 44 vs. Day 61
Wound Closure	0.01*	0.049*	1.0	1.0	1.0
Wound Inflammation	0.005*	0.086	0.639	1.0	1.0
Composite Score	< 0.001*	0.009*	1.0	1.0	1.0

*Represents statistical significance of *p* < 0.05

### Swim performance

**Fig. 5:**
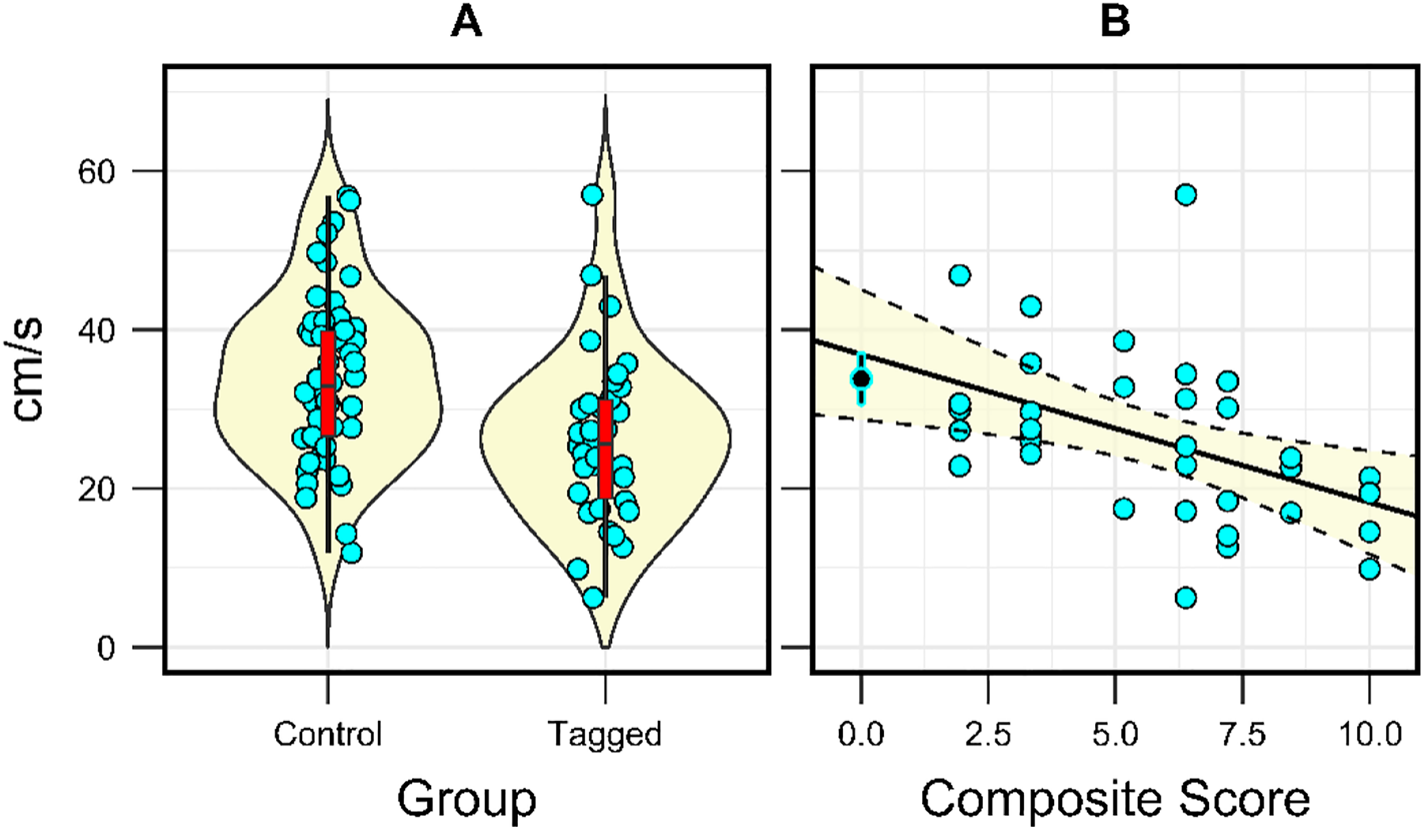
**A** Violin plots of recorded maximum burst speeds for control and tagged sea lamprey, overlaid with box plots exhibiting median (red, black bar), 25% and 75% quartiles (boxes) and non-outlying maximum and minimum speeds (lines, within 1.5 × interquartile range). The raw data are jittered on the X-axis to ease interpretation. **B** Scatterplot and regression of the maximum burst swim speed vs. Day 4 composite wound score. For reference, the mean (± 95% CI) maximum burst speed for the control group is plotted at a value of 0 on the x-axis (black), but was not included in the regression analysis

Burst speed differed between control and tagged groups (ANOVA: p = 0.003, *F* = 9.51), but was not related to origin of the animals (Great Lakes vs Atlantic drainages; *p* = 0.33, *F* = 0.9). Mean maximum burst speed in cm/s (± 1 SE) was 26.22 ± 1.81 for the tagged group and 33.83 ± 1.42 for the untagged group (Fig. 5). The greatest burst speed recorded in each group was 57.02 cm/s for tagged animals and 56.88 cm/s for control animals.

In the tagged group, condition and body size were not related to maximum burst swim speed (linear regression: condition factor, *p* = 0*.*36; total length, *p* = 0.36; mass, *p* = 0.97). There was a significant negative correlation between observed maximum burst speed and each of the wound scores recorded on Day 4 post-surgery (linear regression: wound closure, *p* = 0.031, adjusted *R*^*2*^ = 0.12; wound inflammation, *p* = 0.022, adjusted *R*^*2*^ = 0.12; composite wound score, *p* = 0.007, adjusted *R*^*2*^ = 0.18) (Fig. 5B). However, the composite wound score recorded on the day of the burst swim test (Day 20) was not a significant predictor of maximum burst swim speed (linear regression: *p* = 0.22, adjusted R^2^ = 0.02).

**Fig. 6:**
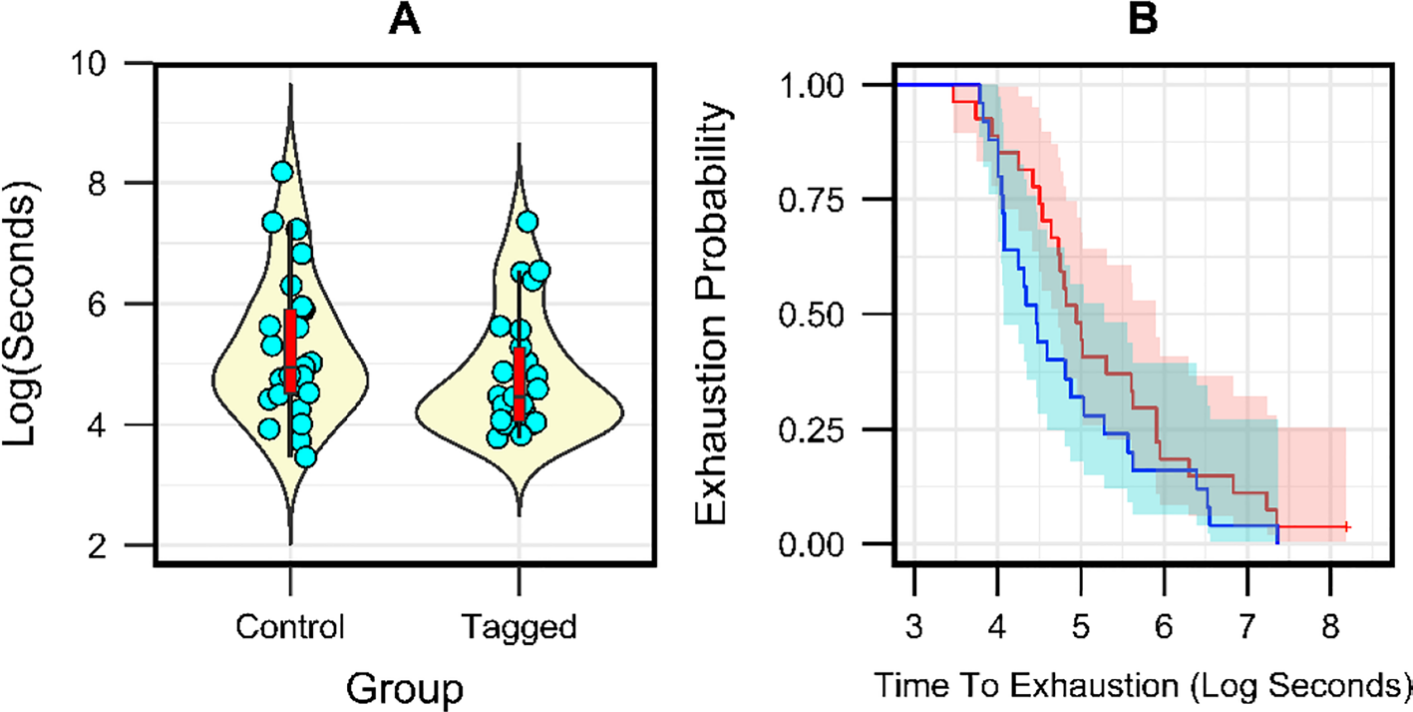
**A** Violin plots of recorded time-to-exhaustion for control and tagged sea lamprey, overlaid with box plots exhibiting median (red, black bar), 25% and 25% quartiles (boxes) and maximum and minimum values for non-outlying results (lines). Data are jittered on the X-axis to ease interpretation. **B** Survival (exhaustion) curves for control (red) and tagged (blue) animals in Swim-to-Exhaustion tests (*p* = 0.18)

Three animals in each of the tagged and control groups could not be induced to swim in the swim tunnel and were not evaluated for swim-to-exhaustion. Two additional animals in each group exhibited lethargy upon retrieval from their holding tank and were censored from the analysis. Sample sizes used in analysis were *n* = 25 tagged and *n* = 27 control. One animal from the control group completed the full trial without exhausting, whereas all other animals in both groups exhausted prior to 60 min. Time-to-exhaustion did not significantly differ between control and tagged groups (ANOVA: *p* = 0.32, *F* = 1.02) and was not related to origin of the animals (Great Lakes vs Atlantic drainages; *p* = 0.17, *F* = 1.96). Median time-to-exhaustion in min. for tagged and control groups were 1.45 (Range = 0.73 – 26.28) and 2.35 (Range = 0.53–60), respectively (Fig. 6A). Similarly, no significant difference in the Kaplan–Meier survival curves was detected (log-rank test, *p* = 0.18; Fig. 6B).

## Discussion

In telemetry studies of juvenile or small fishes, negative impacts of the transmitter on behavior, swim performance, and physiology may result in erroneous conclusions regarding movement trends, behavioral tendencies, and survival rates. Accurate estimation of these components for out-migrating juveniles is critical to understanding the efficacy of conservation measures, such as the installation of fish passage devices and the restoration of spawning and rearing habitats. The results of this study suggest implantation of an ELAT transmitter into newly transformed sea lamprey results in survival and tag retention akin to that reported for similar sized PIT tags. The 61-day survival of our tagged subjects was 71%, well within the range reported in previous studies with larval and juvenile lampreys 25–100%; [14, 29, 49–51, 63]. However, survival was significantly lower in the tagged animals (vs control), with no effect statistically attributable to measures of animal length, mass, condition, or population of origin (Great Lakes vs. Atlantic drainage). There was some evidence that individuals with higher tag burdens were more likely to experience mortality, but given the small sample sizes, that finding should be interpreted with caution.

The significant time-dependent covariate in the Cox model and visualization of the progressive *p-*values suggest the statistical difference between the two groups was heavily weighted by mortalities occurring in the first four days of the experiment. Three lines of evidence suggest the immediate post-surgical mortalities and tag losses were likely caused by imperfect surgical technique, the acute stress of the surgery, or both. First, mortalities and tag losses were distributed across a range of body size, a pattern consistent with a surgeon/surgery effect rather than the consequences of tag internalization [4, 10]. Prior work noted nicking the gastrointestinal tract or other internal organs, and/or protrusion of the intestine through the incision, was associated with rapid mortality (< ~ 7 days) in juvenile lampreys [13, 49, 63] as observed here. Although we did not measure it explicitly, we observed visual evidence of discoloration around the wound site that is consistent with descriptions of internal hemorrhaging provided by Dawson et al. [13, 14]. Two of 5 animals experiencing early mortality in this study exhibited protruding intestines and visual evidence of internal hemorrhaging near the wound site. Second, all tagged animals that survived the four days post-surgery also survived the following two weeks leading up to the first swim test and exhibited clear improvement in wound condition. Third, the time to stage-IV anesthesia was long relative to other anesthetics used with lampreys, and longer than recommendations of all anesthetics (Summerfelt & Smith 1990). Data reported by Christiansen et al. [7] regarding Pacific lamprey transformers suggest a 70 mg/L dose of AQUI-S should result in full sedation in six to nine minutes, we observed a mean time to Stage-IV sedation of 17 min. Strikingly, the time to recovery reported by Christiansen et al. (3–8 min) was considerably faster than we observed (a mean of 102 min). Because our goal was to investigate the use of the ELAT in field studies, our holding tanks and anesthetic bath were maintained at the typical water temperature experienced by an out-migrating transformer in Great Lakes streams (4.5 °C), a temperature substantially colder than reported by Christiansen et al. (12° C). In fishes, colder water temperature can substantially increase the time to uptake and recovery from various anesthetic agents, leading to increases in the rate of post-surgical complications and death [64, 77]. Our results suggest that use of alternative anesthetics (e.g., MS-222) should be considered when performing surgeries in cold water on juvenile sea lamprey. In addition, our study was required to follow Federal Investigational New Animal Drug (INAD) guidelines for the use of AQUI-S (i.e., dosage and exposure time) contained in INAD #11-741 [71]. At a minimum, anesthesia management procedures for cold-water use of AQUI-S need to be better developed for use in juvenile sea lamprey. Future studies with greater availability of subjects should also include anesthesia-only and anesthesia+surgery sham controls to better disentangle the relative effects of each factor in survival and performance tests.

It is noteworthy that wound healing was slower in our subjects when compared to the two available studies of PIT tagging transforming or transformed juvenile lampreys despite a similar incision size (3 mm). In the present study, one surviving tagged fish exhibited a fully closed wound at the conclusion of the 61-day study period (2.2%), with the majority of improvement in the wounds occurring between Days 4 and 20. Simard et al. [63], studying metamorphosing anadromous (Fort River, MA) and land-locked (Lake Champlain) sea lamprey, report post-surgical skin closure rates of approximately 15–20% after 60 days of healing, though rates varied across populations (12 mm PIT tag surgically inserted, ~ 5 °C). Mesa et al. [46] report only 2.2% of transformed Pacific lamprey held at 9–18 °C exhibited signs of poorly healed or infected incisions 40 days following implantation of a 9 mm PIT tag. At Day 20, our results were akin to those reported by Moser et al. [49] for Pacific lamprey ammocoetes tagged with an 8.4 mm PIT tag and held in the field for 15 days at 6– 11 °C (i.e., no healed wounds). Despite frequent reports of fungal infections in other laboratory studies using juvenile lampreys [46, 50, 61, 63], infections did not arise in our subjects. The water temperature used here was predominantly lower than that of the studies mentioned, in most cases by > 4 °C (~ 5–18 °C); thus, the use of metamorphosed animals held at low water temperature may have both slowed the rate of wound healing and inhibited fungal infection. However, it is likely that use of this transmitter in out-migrating lamprey within Great Lakes watersheds will involve animals experiencing similar water temperatures. Slow healing may be a significant concern in field studies, where more vigorous swimming and burial behaviors may further slow wound closure and may also increase the likelihood of tag loss (vs. that observed in tank studies). Moser et al. [49] report slower healing for juvenile Pacific lamprey held in a natural stream vs. those held in laboratory tanks. Options for defending against this outcome in field studies include the addition of a suture to hasten wound closure and better retain the transmitter, performing surgeries under warmer water in the laboratory (with appropriate acclimation periods), or simply targeting studies to warmer times of year.

Implantation with an ELAT transmitter negatively affected swim performance. Maximum burst swim speed in tagged animals was 7.6 cm/s (22%) less than untagged animals, regardless of source population, suggesting a substantial reduction in the ability to perform high-speed acceleration. Interestingly, the composite wound score taken four days post-surgery was a significant predictor of burst swim speed measured more than two weeks later, whereas the score on the day of the test was not. Further, the predicted burst swim speed for a wound score of zero (completely healed) was 36.9 cm/s, similar to the mean burst speed observed in untagged animals (33.8 ± 1.4 cm/s). Mueller et al. [50] report a 6 cm/s reduction in the burst swim speed of actively out-migrating Pacific lamprey, although the relative reduction vs. untagged animals (7%) was substantially lower than in our study, despite using similarly sized subjects. The difference is largely attributable to that the recorded burst swimming speeds of Pacific lamprey are much higher which appears consistent across studies (means of 51–76 cm/s), e.g., [12, 67]. It is important to note that these studies were also performed at higher temperatures (12 and 20 °C, respectively) and that swim performance may be strongly affected by temperature in fishes [6, 74, 75]. Conversely, and despite a 38% decrease in median value, we failed to detect a significant negative effect of the transmitter in the time-to-exhaustion swim test. Why the effect of the tag manifested more strongly during burst vs. steady swimming is of interest, as burst or “escape” swimming is more associated with successful passage through hydraulic challenges and the avoidance of predators during out-migration [21]. When a juvenile sea lamprey accelerates from rest, it generates a high amplitude body bend (double that of steady swimming) that propagates over 75% of the body length [20]. It is plausible there is an amplitude threshold where the transmitter becomes impinged on the viscera, restricting the ability to generate thrust, and aggravating any internal injury associated with the tag implantation surgery. That threshold is more likely to be breached during high acceleration swimming, and conceivably may affect the estimation of natural mortality rates in field studies with small, narrow-bodied anguilliform fishes.

A core assumption in telemetry studies is that tagged individuals are characteristic of the natural population in terms of size, sex, movement characteristics, and fate [30]. Our experimental animals were somewhat smaller than the historic mean size observed in the Great Lakes, and did not encompass the full size range of available fish. Although we did not observe significant statistical effects of animal size on the measured outcomes, the surgeon reported difficulty inserting the transmitter into subjects with smaller body cavities, and there was some evidence that individuals with tag burdens in excess of two percent of body mass suffered greater mortality. Several lamprey also exhibited light discoloration posterior to the implanted tag soon after surgery, a symptom of blood impediment through the dorsal aorta caused by pressure from the tag [13] that appeared prior to recovery from anesthesia. However, it was not a consistent indicator of impending mortality. Two of 5 animals displaying this symptom no longer exhibited discoloration by the first wound assessment on Day 4 and survived the length of the study, perhaps due to a posterior shift in tag position as posited by Moser et al. [49]. It was also notable that in both swim tests there was substantial variation in performance among individuals that was unrelated to measures of body size or population of origin. A number of studies demonstrate both burst and steady swimming speeds, and the underlying aerobic scope for activity, are repeatable traits within individual fish during swim performance tests, e.g., [8, 23, 36, 52]. Understanding consistent individual differences in tag effects on swim performance is likely an important metric to understanding whether and how the effects of the transmitter on swimming will manifest in studies designed to measure movement rates and survival in the wild [73]. Further, out-migrating juvenile lampreys exhibit strong nocturnal patterning in downstream movements, burrowing into sediment or seeking shelter during daylight hours [12, 47, 54]. In a study with smaller (means of < 120 mm) larval sea lamprey implanted with 8 or 9 mm PIT tags, the tag significantly increased both the time (2.3 × longer) and effort (defined as number of “stops” while burrowing, 1 additional stop on average) necessary for the animal to successfully burrow (vs. untagged fish; [13]).

It is routine for tag effect studies to recommend minimum size limits for use of animals in telemetry work, based on the desire to satisfy the criteria of minimal tag effect on the animal’s performance. However, this practice conflicts with the need to use representative animals, as demographic measures, such as mortality, are highly size dependent in juvenile fishes [2, 65]. In other words, preferential use of larger subjects that are less likely to suffer tag effects (i.e., non-probability sampling) may bias estimates of vital rates made using those animals in field studies. Conceivably, this may be compensated in the modeling of mortality rates from telemetry studies by using size-dependent estimates of tag-induced mortality. Similar studies to ours in larval and juvenile Pacific and sea lamprey offer minimum size recommendations of 120–150 mm total length, based on the relationship between mortality and length observed in the lab [46, 50, 61, 63]. While there was no significant correlation of any size measurement and mortality in our tagged animals, larger mass, but not total length, was associated with an improved composite wound score, suggesting lesser injury during the surgery. Post-metamorphosis, the intestines of a sea lamprey range laterally throughout the body of the animal, lessoning the available space between the viscera and the body wall [42]. We suggest that body girth at the tag location, or a similar metric (e.g., ratio of body depth to length), or a measure of condition factor, may prove more suitable metrics to set the minimum threshold for implantation. These metrics require a larger dataset, perhaps integrated across previous and future studies to solidify a concrete threshold. Our findings support a ratio of 3.33:1 (length (mm) to mass (mg)), above a minimum length and mass of 150 mm and 45 mg, should ensure high survival. We further recommend a brief holding period (2–4 days) after recovery from anesthesia and prior to release to assess wound condition and potential early tag loss. If the wound does not appear to have inflammation over a moderate level and/or a complete lack of apposed tissue around the incision, it is likely that the animal will experience limited physical incapacitation compared to an untagged conspecific. Additionally, although not experienced in our study, prior work with juvenile lampreys suggest fungal infection is a risk whenever the fish are held in tanks, especially at warmer temperatures. A prophylactic anti-fungal regime may be needed in those conditions.

## Conclusions

The ELAT transmitter has now proven feasible in laboratory and field settings across a range of anguilliform species with relevant behavioral unknowns, such as the regionally endangered American brook lamprey (*Lethenteron appendix*) of the United States Eastern Seaboard and the critically endangered European eel (*Anguilla Anguilla*) (Department, N.H. Fish and Game [18], The IUCN Red List of Threatened Species [68]) [16, 17, 39]. Our findings demonstrate its utility for juvenile sea lamprey. The ability to gain insight into the fate of migrants as a function of the features of the rivers that regulate mortality (e.g., predator density, habitat type, etc.) is likely to lead to the development of new control methods to target sea lamprey in their last phase before the initiation of parasitism [33]. In addition, it allows for the investigation of the effects of habitat degradation and barriers to downstream migration (dams and weirs, water intake structures), and overharvesting; the principal threats to several migratory lampreys in their native range [28, 35, 40, 44, 48]. Ultimately, the ELAT transmitter has the potential to open the door to a greater understanding of the out-migration parasitic lampreys, a critical and poorly understood stanza in the lives of these consequential and enigmatic creatures.

## Data Availability

All datasets used in this publication are available upon request to the corresponding author.

## Abbreviations

ELAT: Eel–lamprey acoustic transmitter
PIT: Passive Integrated Transponder
JSATS: Juvenile Salmon Acoustic Telemetry System
PRI: Pulse Rate Interval
USFWS: United States Fish & Wildlife Service
INAD: Investigational New Animal Drug
TDC: Time-Dependent Covariate
AIC: Akaike Information Criterion
